# Expression profiling and bioinformatic analyses suggest new target genes and pathways for human hair follicle related microRNAs

**DOI:** 10.1186/s12895-017-0054-9

**Published:** 2017-02-22

**Authors:** Lara M. Hochfeld, Thomas Anhalt, Céline S. Reinbold, Marisol Herrera-Rivero, Nadine Fricker, Markus M. Nöthen, Stefanie Heilmann-Heimbach

**Affiliations:** 1 Institute of Human Genetics, University of Bonn, Sigmund-Freud-Str. 25, 53127 Bonn, Germany; 2 Department of Genomics, Life and Brain Center, University of Bonn, Sigmund-Freud-Str. 25, 53127 Bonn, Germany; 3 Human Genomics Research Group, Department of Biomedicine, University of Basel, Hebelstrasse 20, 4031 Basel, Switzerland

**Keywords:** miRNA, mRNA, Gene regulation, Human hair biology, Correlation analysis

## Abstract

**Background:**

Human hair follicle (HF) cycling is characterised by the tight orchestration and regulation of signalling cascades. Research shows that micro(mi)RNAs are potent regulators of these pathways. However, knowledge of the expression of miRNAs and their target genes and pathways in the human HF is limited. The objective of this study was to improve understanding of the role of miRNAs and their regulatory interactions in the human HF.

**Methods:**

Expression levels of ten candidate miRNAs with reported functions in hair biology were assessed in HFs from 25 healthy male donors. MiRNA expression levels were correlated with mRNA-expression levels from the same samples. Identified target genes were tested for enrichment in biological pathways and accumulation in protein-protein interaction (PPI) networks.

**Results:**

Expression in the human HF was confirmed for seven of the ten candidate miRNAs, and numerous target genes for miR-24, miR-31, and miR-106a were identified. While the latter include several genes with known functions in hair biology (e.g., *ITGB1, SOX9*), the majority have not been previously implicated (e.g., *PHF1*). Target genes were enriched in pathways of interest to hair biology, such as integrin and GnRH signalling, and the respective gene products showed accumulation in PPIs.

**Conclusions:**

Further investigation of miRNA expression in the human HF, and the identification of novel miRNA target genes and pathways via the systematic integration of miRNA and mRNA expression data, may facilitate the delineation of tissue-specific regulatory interactions, and improve our understanding of both normal hair growth and the pathobiology of hair loss disorders.

**Electronic supplementary material:**

The online version of this article (doi:10.1186/s12895-017-0054-9) contains supplementary material, which is available to authorized users.

## Background

The human hair follicle (HF) passes through cycles of active growth (anagen); regression (catagen); and rest (telogen). Each of these stages is tightly regulated, and is characterised by distinct changes in gene expression, cell proliferation, and differentiation [1, 2].

Micro(mi)RNAs are short (~20-25 nucleotides), non-coding RNAs, which influence gene expression by binding to target messenger(m)RNAs via a complementary seed region, which elicits mRNA degradation or transcriptional inhibition. In recent years, accumulating research data have indicated the importance of miRNAs as potent regulators of numerous developmental and pathobiological processes [3]. Several miRNAs have been implicated in hair biology, e.g., in the control of hair pigmentation, HF cycling, and keratinocyte differentiation [4–6]. For instance, miR-137 is reported to be responsible for coat colour determination in mice [5], while the inhibition of miR-31 in murine skin has been shown to result in accelerated anagen progression and abnormal hair shaft morphology [4]. A further study reported, a differential expression for four miRNAs (miR-106a, miR-410, miR-221, miR-125b) in dermal papilla cells (DPCs) from the balding and non-balding scalp areas of eight patients with male pattern baldness (MPB) [7]. However, the majority of available data on the role of miRNAs in hair biology have been obtained from mouse or cell culture experiments, and knowledge of the genes and pathways that are targeted by these miRNAs in the human HF is limited. Such knowledge is essential in terms of understanding the relevance of miRNAs to human hair (patho-) biology.

The aims of the present study were to: 1) perform a systematic investigation of the expression of ten candidate miRNAs (miR-22, miR-24, miR-31, miR-106a, miR-125b, miR-137, miR-205, miR-214, miR-221, miR-410) in human HF samples; 2) correlate these data with corresponding HF mRNA expression levels; and 3) test the identified target genes for enrichment in pathways and protein networks in order to delineate regulatory interactions in the human HF.

## Methods

### Sample collection and nucleic acid extraction

HF samples were collected from the frontal- and the occipital scalp areas of 25 volunteer healthy male donors of European descent (mean age 24.2 years ± 1.6). RNA and miRNA were extracted from HF tissue using the miRNeasy Mini Kit and the RNeasy^®^ MinElute^®^ Cleanup Kit (Qiagen, Hilden, Germany). The quantity and quality of the extracted RNAs and miRNAs were tested on an ND-1000 spectrophotometer (Peqlab Biotechnologie, Erlangen, Germany) and a BioAnalyzer 2100 (Agilent Technologies, Waldbronn, Germany), respectively. Samples with an RNA concentration of ≥20 ng/μl, an RNA integrity number (RIN) of ≥8 and a miRNA concentration of ≥25 ng/μl were included in the microarray analysis.

### miRNA profiling

MiRNA profiling of n = 50 samples (25 frontal, 25 occipital) was performed on the Affymetrix^®^ GeneChip^®^ miRNA 4.0 (Affymetrix, Santa Clara, CA) using a total of 250 ng of HF miRNA. Poly(A) tailing and biotinylation were performed with the Affymetrix^®^ GeneChip^®^ Hybridization, Wash, and Stain Kit, in accordance with the manufacturer’s instructions. After scanning, miRNA raw expression values were background subtracted, quantile normalised and log_2_-transformed using robust multi-array analysis (RMA) and detection above background (DABG) in the Affymetrix^®^ Expression Console™ (Affymetrix Santa Clara, CA). A total of 48 samples from 24 individuals fulfilled all quality control criteria. Candidate miRNAs were considered to be expressed if they were defined as ‘present’ in ≥80% of all samples.

### mRNA profiling

Whole transcriptome profiling of the corresponding HF RNA samples was performed using the TotalPrep™-96 RNA Amplification Kit and Illumina HT-12v4 Bead Arrays (Illumina Inc., San Diego, CA). Background subtracted expression intensities and detection *P*-values were exported from the Illumina GenomeStudio software. These were then quantile normalised and log_2_-transformed using the R package ‘limma’. Only probes with all of the following four characteristics were taken into account: (i) a detection *P*-value of <0.05 (indicating significant expression above background) in at least 80% of the samples; (ii) a good or perfect probe quality; (iii) an annotated Entrez gene identifier, as reported in the Bioconductor package illuminaHumanv4.db [8]; and (iv) no single nucleotide polymorphism within the probe sequence (dbSNP Build 142). After filtering, a total of 10,029 expression probes, corresponding to 8,210 gene symbols, remained for the correlation analysis.

### Selection of candidate miRNAs

Candidate miRNAs were selected based on the results of a comprehensive PubMed literature search for the role of miRNAs in HF biology. A total of ten candidate miRNAs (miR-22, miR-24, miR-31, miR-106a, miR-125b, miR-137, miR-205, miR-214, miR-221, and miR-410) were selected for investigation in the human HF (PMIDs: 26020521, 20522784, 21362569, 21967250, 22847819, 23974039, 24232098, and 25422376). These miRNAs were represented by 21 expression probes on the Affymetrix miRNA4.0 array (Table 1).

**Table 1:** Overview of selected candidate miRNAs: Previously reported role(s) in hair biology and expression status of the analysed miRNAs in the human hair follicle (HF)

Candidate miRNA	Reported role in hair biology	Reference	Mature form on miRNA array	HF Expression	# of uniquely correlated genes
miR-31	Inhibits anagen development by regulating gene expression programmes and alters hair shaft formation in mice	Mardaryev AN et al., 2010	hsa-miR-31-5p	✓	99
			hsa-miR-31-3p	✓	-
miR-24	Overexpression is associated with reduced proliferation and premature HF-keratinocyte differentiation in mice	Amelio I et al., 2013	hsa-miR-24-3p	✓	103
			hsa-miR-24-1-5p	✓	-
			hsa-miR-24-2-5p	✓	5
miR-106a	Upregulated in balding human DPC in comparison to nonbalding DPCs	Goodarzi HR et al., 2012	hsa-miR-106a-5p	✓	53
			hsa-miR-106a-3p	✗	-
miR-22	Overexpression in mice is associated with hair loss due to anagen-to-catagen transition and knockout in mice is associated with delayed catagen entry and accelerated telogen-to-anagen transition	Yuan S et al., 2015	hsa-miR-22-5p	✓	-
			hsa-miR-22-3p	✓	
miR-125b	Represses HF stem cell differentiation in mice; significantly upregulated in balding human DPCs in comparison to nonbalding DPCs	Zhang L et al., 2011; Goodarzi HR et al., 2012	hsa-miR-125b-5p	✓	–
			hsa-miR-125b-1-3p	✗	
			hsa-miR-125b-2-3p	✓	
miR-137	Involved in murine HF pigmentation (melanogenesis)	Dong C et al., 2012	hsa-miR-137	✗	–
miR-205	Essential for development of HF stem cell proliferation during murine embryonic skin development	Wang D et al., 2013	hsa-miR-205-5p	✓	–
			hsa-miR-205-3p	✓	
miR-214	Controls Wnt pathway and β-catenin expression in murine embryonic HF development	Ahmed MI et al., 2014	hsa-miR-214-5p	✗	–
			hsa-miR-214-3p	✗	
miR-221	Upregulated in balding human DPCs in comparison to nonbalding DPCs	Goodarzi HR et al., 2012	hsa-miR-221-5p	✓	–
			hsa-miR-221-3p	✓	1
miR-410	Upregulated in balding human DPC in comparison to nonbalding DPCs	Goodarzi HR et al., 2012	hsa-miR-410-5p	✗	–
			hsa-miR-410-3p	✗	

*HF* hair follicle, *DPCs* dermal papilla cells, *#* number

### Target gene identification

To identify targets genes, mean miRNA and mRNA expression levels were calculated from the frontal and occipital sample of each of the final 24 participants. The expression levels of 10,029 mRNA probes and seven expressed candidate miRNAs which were represented by 14 mature miRNA forms were correlated using the Pearson correlation analysis method [9]. The respective correlation coefficients (*r*) were computed and the resulting *P*-values were then corrected for multiple testing using Benjamini-Hochberg correction (*P*_adj_). All mRNAs with a significant correlation (*P*_adj_ <0.01) to a candidate miRNA were assumed to be target genes. To exclude correlations driven by differential expression between frontal and occipital samples, the correlation trend was confirmed via single-tissue analysis (Additional file 1: Table S1, Additional file 2: Figure S1).

### Pathway enrichment of, and protein-protein interactions (PPIs) between, miRNA target genes

For all significantly correlated target genes, testing for pathway enrichment was performed using the Ingenuity Pathway Analysis software (IPA, Qiagen, Hilden, Germany, accessed 31 March 2016); and the Protein ANalysis THrough Evolutionary Relationship database (PANTHER, version 11.1, http://pantherdb.org/, accessed 2nd December 2016) [10]. Only pathways with ≥3 annotated genes and a *P*-value based on right tailed Fisher’s exact test <0.05 (IPA) were taken into account.

PPIs were investigated using the Search Tool for the Retrieval of Interacting Genes/Proteins (STRING, version 10, http://string-db.org, accessed 2nd December 2016) [11].

### miRNA target prediction

The miRWalk2.0 (http://zmf.umm.uni-heidelberg.de/apps/zmf/mirwalk2, accessed 11 March 2016) [12]; and the TargetScan7.0 (http://targetscan.org, accessed 2nd December 2016) [13] algorithms were used to search for predicted and validated target genes of the expressed candidate miRNAs. Only genes that were predicted by the miRWalk algorithm and three additional implemented databases, or that were predicted to target a conserved site in TargetScan, were taken into account.

## Results

Seven of the ten candidate miRNAs were expressed in both the frontal an occipital HF samples. The strongest mean log_2_ expression (log_2__value) was found for miR-205 (log_2__value = 3.73 ± 0.01), and miR-24 (log_2__value = 3.69 ± 0.03). Using the present criteria, no expression was observed for miR-137 (log_2__value = -1.77 ± 1.72); miR-214 (log_2__value = 1.50 ± 0.56); or miR-410 (log_2__value = -1.08 ± 0.61) (data not shown).

**Fig. 1:**
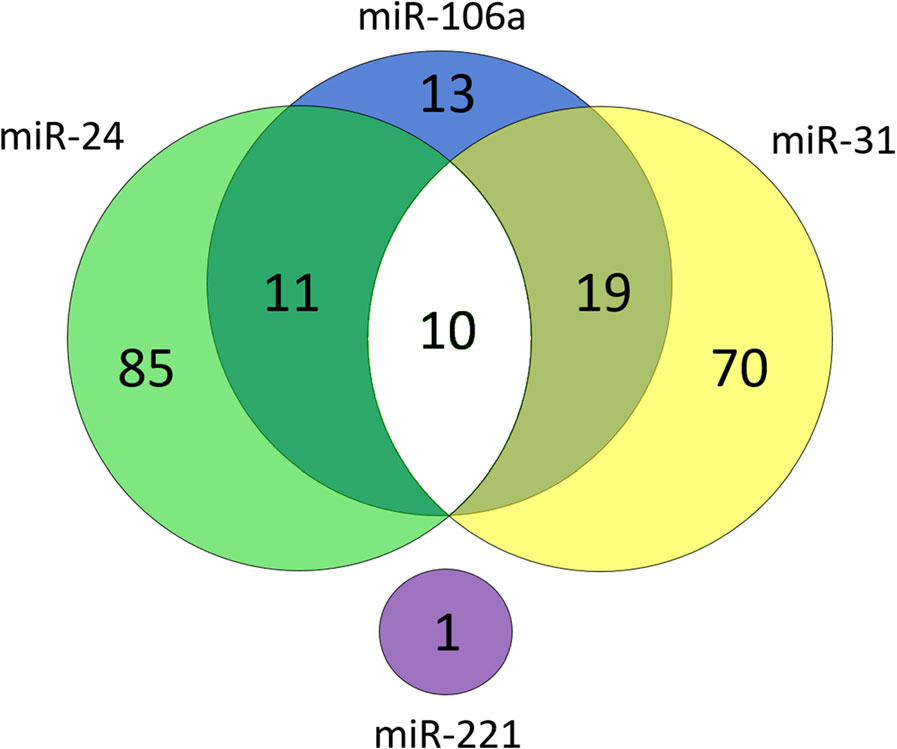
Overview of all target genes with a significant correlation to miR-24, miR-31, and miR-106a. The largest overlap in target genes was detected for miR-31 and miR-106a (n = 29). MiR-31, miR-24 (i.e., miR-24-3p, miR-24-2-5p), and miR-106a shared the following ten target genes: *FZD7, JUN*, *MEIS2*, *TAX1BP3, RBM17*, *SFRP1*, *TP63*, *SMARCA4, COL17A1,* and *ZCCHC11*. The same ten target genes were shared between miR-31 and miR-24. MiR-24 and miR-106a shared a total of 21 target genes. No overlap was found for miR-221 and the three remaining miRNAs

To investigate the function of these seven miRNAs, and to identify their target genes in the human HF, a correlation analysis of intrasample mean miRNA and mean mRNA expression was performed. Significant correlation between miRNA and mRNA expression was observed for miR-24, miR-31, miR-106a, and miR-221. For miR-24 (i.e., miR-24-3p, miR-24-2-5p), a significant correlation was found with 106 genes: *n* = 74, negatively correlated (neg. cor.); and n = 32, positively correlated (pos. cor.). The two most significantly correlated genes were *COL5A2* (Collagen, Type V, Alpha 2; *r* = -0.92, *P*_adj_ = 1.70 × 10^-5^); and *SERPING1* (Serpin Family G Member 1*; r* = -0.86, *P*_adj_ = 8.77 × 10^-4^). For miR-31, a total of 99 genes (53 neg. cor. and 46 pos. cor.) were identified. Here, the two most significantly correlated genes were *FAM178A* (SMC5-SMC6 complex localisation factor 2; *r* = -0.90, *P*_adj_ = 1.51 × 10^-4^); and *PLAA* (Phospholipase A2-Activating Protein; *r* = 0.89, *P*_adj_ = 1.82 × 10^-4^). MiR-106a expression was correlated with a total of 53 genes (29 neg. cor. and 24 pos. cor.). Here, the two most significantly correlated genes were *UST* (Uronyl-2-Sulfotransferase; *r* = -0.86, *P*_adj_ = 8.77 × 10^-4^); and *COL5A2 (r* = -0.85, *P*_adj_ = 8.77 × 10^-4^) (Additional file 1: Table S1). For miR-221, correlation was found with a single gene - *RPRD2* (Regulation Of Nuclear Pre-MRNA Domain Containing 2; *r* = -077. *P*_adj_ = 7.59 × 10^-3^). A total of 40 genes were targets of more than one miRNA. The largest overlap was found between target genes of miR-31 and miR-106a (n = 29). Ten genes (*FZD7*, *JUN*, *MEIS2*, *TAX1BP3*, *RBM17*, *SFRP1*, *TP63*, *ZCCHC11*, *COL17A1, SMARCA4*) were significantly correlated with miR-24, miR-31, and miR-106a (Fig. 1, Additional file 1: Table S1).

In the investigation of a potential enrichment of miRNA target genes in biological pathways, IPA revealed the strongest enrichment of the respective target genes in ‘Hepatic Fibrosis/Hepatic Stellate Cell Activation’ (miR-24), and ‘JAK/STAT Signalling’ (miR-31 and miR-106a). In the PANTHER analysis, ‘Integrin Signalling’ was the top pathway for the target genes of miR-24, miR-31 and miR-106a. An overview of all identified pathways is provided in Additional file 1: Table S2.

In the miRWalk2.0 [12] and TargetScan7.0 [13] analyses, 40%, 62%, and 42% respectively of the identified target genes for miR-24, miR-31 and miR-106a were not predicted by either tool. The single target gene of miR-221 was predicted by miRWalk only (Additional file 1: Table S3).

**Fig. 2:**
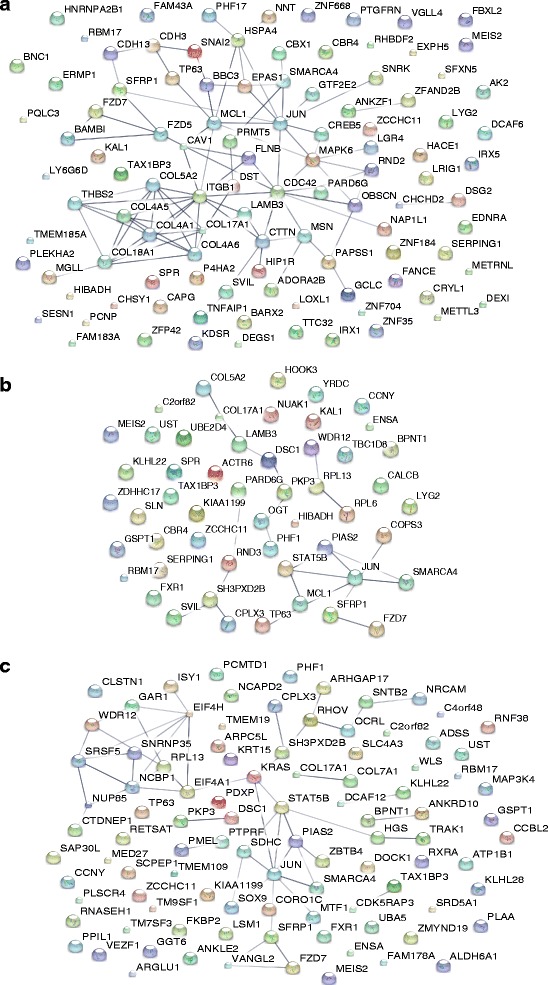
STRING protein-protein interaction (PPI) query. PPIs of significantly correlated target genes **a** miR-24; **b** miR-106a; and **c** miR-31. Connecting lines represent confidence interactions according to the STRING database. The genes *JUN*, *SFRP1,* and *FZD7* were targets of all three miRNAs and show a consistent PPI in combination with other miRNA-specific target proteins

The STRING [11] database query revealed numerous interactions between the miRNA-specific-, shared- and all target genes of all four miRNAs. In all PPI-networks, an interaction was observed between JUN and FZD7 via SFRP1 (Fig. 2, Additional file 2: Figure S2).

## Discussion

The present study involved comprehensive analysis in the human HF of ten miRNAs previously implicated in hair biology [7, 14]. Expression profiling confirmed the expression of seven of the ten candidate miRNAs, suggesting that these miRNAs may indeed play a role in human hair biology. For miR-24, miR-31, and miR-106a several target genes and pathways of interest were identified (Table 1).

The highest number of target genes was identified for miR-24. Previous research has identified miR-24 as an anti-proliferative miRNA, which promotes keratinocyte differentiation via the modulation of actin filaments [15], and plays a role in hair morphogenesis [6]. For miR-24 (i.e., miR-24-3p, miR-24-2-5p), correlation analysis revealed a total of 106 unique target genes. These include the miRWalk2.0 predicted target *ITGB1,* which encodes the integrin β-1 subunit and has been subject to extensive investigation with respect to skin and hair homeostasis (reviewed in Rippa *et al.*, 2013 [16]). The present pathway analysis also revealed an enrichment of miR-24 target genes in ‘Integrin Signalling’. These results suggest that integrin signalling is an essential pathway for keratinocyte differentiation in the human HF, and that this is controlled by miR-24. Furthermore, significant correlations with miR-24 expression were observed for six collagen genes. In descending order of significance, these were: *COL5A2*, *COL17A1*, *COL4A6*, *COL4A5*, *COL18A1* and *COL4A1.* The respective gene products also form a dense PPI-network (Fig. 2). Previous functional studies have demonstrated hair coat thinning and abnormal HF morphogenesis in mice that overexpress miR-24 in basal keratinocytes. These mice display shorter, misangled, and wavy HFs [6]. A similar hair phenotype is seen in patients with the chromosome 2q32 deletion syndrome, whose clinical features include thin, sparse, woolly, and slowly growing scalp hair [17, 18]. Interestingly, the affected 2q32 chromosomal region includes *COL5A2*. Another collagen gene, *Col17a1,* is reported to be essential for HF stem cell maintenance [19] and age-associated HF miniaturisation and thinning, as mediated by COL17A1 proteolysis [20]. Moreover, *COL17A1* deficiency is associated with junctional epidermolysis bullosa, a severe skin disease characterised by hair loss [21]. Taken together, these data suggest that miR-24 is an important regulator of hair morphogenesis and maintenance, which achieves its effect via the control of integrin and collagen signalling. The present study also detected an enrichment of miR-24 target genes in the hormone signalling cascades ‘Gonadotropin Releasing Hormone (GnRH) Receptor Pathway’, and ‘Androgen Signalling’. Whereas androgen signalling is essential for hair biology and has been shown to regulate hair growth and cycling at different body sites [22], GnRH signalling antagonises androgen receptor signalling at androgen-sensitive body sites in women, and GnRH antagonists are an effective treatment for hirsutism [23, 24]. Research is warranted to determine whether these hormone pathways also play a role in keratinocyte differentiation.

Research has shown that miR-31 is responsible for both anagen inhibition and normal hair shaft formation [4]. The present analyses identified a total of 99 target genes that may act downstream of miR-31 in these processes. These include Retinoid X Receptor Alpha (*RXRA*), a nuclear receptor which is highly expressed in skin and in HF outer root sheath (ORS) keratinocytes [25, 26]. In mice, ablation of *Rxra* in the skin leads to HF degeneration and subsequent hair loss [27], while conditional knockout in epidermal and ORS keratinocytes results in altered anagen initiation [28]. These findings underline the role of *RXRA* in HF maintenance and hair cycle control. Interestingly, target genes of miR-31 were enriched in *PPAR* and *RAR/RXRA* signalling, thus supporting the hypothesis that *RXRA*-mediated signalling is important for the control of anagen initiation. Moreover, miR-31 target genes were enriched in PDGF (Platelet-Derived Growth Factor), adipogenesis, and JAK/STAT signalling, which have been implicated previously in the control of the HF cycle [29–33]. Studies in murine HFs have demonstrated that several PDGF isoforms induce and maintain murine anagen HFs [31]. Furthermore, PDGF signalling may contribute to the essential role of immature adipocytes in anagen induction [34]. A recent study identified JAK/STAT signalling as a promising therapeutic target for the treatment of hair loss disorders. Here, topical application of JAK/STAT inhibitors to the shaved back skin of mice led to rapid anagen induction [33]. Collectively, the identified target genes and pathways indicate that miR-31 is a potent cross-species inhibitor of the anagen phase. However, functional studies are required to confirm the interaction between miR-31 and these pathways, and to elucidate their role in anagen control in the human HF.

The third miRNA to show significant mRNA correlations in the present analyses, miR-106a, is reported to be upregulated in the balding, as compared to the non-balding, DPCs of males with MPB, which suggests that it may be implicated in MPB pathobiology [7]. Although none of the 53 identified target genes of miR-106a have yet been associated with MPB, two building blocks of the desmosome - Plakophilin 3 (*PKP3*) and Desmocollin 1 (*DSC1*), are reported to play a role in HF morphogenesis [35]. *Pkp3* deficient mice develop an abnormal hair coat and secondary alopecia [36]. Although *Dsc1* deficient mice show normal HF cycling and structures until the age of four weeks, they develop alopecia and HF degeneration in later life [37]. Moreover, one of the pathways identified in the present study was ‘WNT Signalling’, which is of key importance in terms of HF development and cycling [38–41]. Interestingly, genetic evidence is available for the involvement of WNT signalling in MPB development. Heilmann et al. reported that a single nucleotide polymorphism (rs7349332) located intronically in *WNT10A* was associated with MPB risk (*P* ≤ 5 × 10^-8^) and resulted in reduced *WNT10A* expression in HFs of risk allele carriers [42]. The present analyses therefore provide strong support for the hypothesis that miR-106a contributes to MPB development via WNT signalling and that the regulation of cell-cell adhesion may be an important factor in MPB.

Intriguingly, ten of the identified target genes were shared between miR-31, miR-24, and miR-106a, suggesting that they may be critical points in the signalling cascades that control HF biology. The overlapping target genes *FZD7*, *SFRP1,* and *TAX1BP3* are involved in WNT/β-catenin signalling, which is an important biological pathway for HF development and maintenance [43–46]. The WNT receptor *FZD7* mediates canonical and non-canonical signalling [47, 48], while *SFRP1* and *TAX1BP3* are reported as WNT antagonists. The respective proteins exert their effects via direct interaction with WNT or FZD proteins [49] and binding to β-catenin [50], respectively. Another interesting shared target gene is *TP63*, since one characteristic of *p63* knockout mice is the absence of HFs [51]. *SMARCA4* is a component of the chromatin remodelling complex, and knock-out experiments in murine bulge cells showed that it is required for hair regeneration and anagen progression [52]. The role of *COL17A1* in hair biology was discussed earlier. The four remaining overlapping target genes have not yet been associated with HF biology. The gene *RBM17* is reported to be involved in mRNA splicing [53], *JUN* belongs to the AP-1 transcription factor family, and is involved in many fundamental cell processes including proliferation, differentiation, and apoptosis and plays an essential role in skin development and the differentiation of epidermal keratinocytes [54–59]. *MEIS2* encodes a TALE homeobox protein, which is a highly conserved transcription factor, and *ZCCHC11* is a zinc finger containing RNA uridyltransferase. Taken together, these results underline the importance of WNT signalling in hair biology and suggest that miRNAs are critical regulators of WNT and TP63 signalling in the human HF.

In addition to these ten shared genes, a total of 30 genes were targeted by two miRNAs. These therefore represent further promising candidate genes, which may impact key functions in healthy hair biology and the pathobiology of hair loss disorders.

According to the STRING database, numerous interactions exist between the identified target genes of each candidate miRNA, and among the 40 shared miRNA target genes. This indicates that these miRNAs are (in)directly involved in various regulatory networks. Notably, all of these networks contain an indirect interaction between *JUN* and *FZD7* via *SFRP1*, suggesting that these genes may play a pivotal role in the miRNA mediated control of HF cycling, keratinocyte differentiation and MPB development (Fig. 2, Additional file 2: Figure S2).

The present analyses failed to confirm the expression of miR-137, miR-214 and miR-410 in the human HF. While miR-137 has been described in the determination of murine coat colour [5], no data are available concerning the expression pattern of miR-137 in the human HF or skin. Research has shown that miR-214 controls WNT/β-catenin signalling in murine embryonic HF development [60]. Further studies are required to determine whether miR-137 is involved in the determination of human hair colour and whether miR-214 plays a role in human HF embryogenesis. MiR-410 expression was found almost exclusively in the DPCs of balding vellus HFs [7]. As the present analyses were restricted to non-balding HFs with sparse DPCs, the failure to detect miR-410 expression in the present samples may point to a very specific role for miR-410 during MPB pathogenesis. Moreover, no significant correlation was found with mRNA expression for miR-22, miR-125b, or miR-205. This may be attributable to limited power of our sample (n = 24) to detect smaller regulatory effects.

## Conclusions

In conclusion, the present systematic investigation of the expression of ten miRNAs previously implicated in hair biology and the identification of their target genes, pathways, and regulatory networks provides novel insights into the biological mechanisms that control human HF cycling, HF keratinocyte differentiation, and MPB pathogenesis. Further analyses in larger samples and detailed functional follow up investigations, such as the precise miRNA localisation in the human HF and their expression profile during different hair cycle stages, are now warranted to confirm these findings and to identify additional target genes and regulatory interactions. Increased sample sizes will also allow genome-wide investigations and thus the identification of additional hair-relevant miRNAs, as well as their target genes and regulated pathways. This research will facilitate understanding of human hair (patho-) biology.

## Additional files

Additional file 1: Tables S1-S4. Significantly correlated target genes of miR-24, miR-31, miR-106a, and miR-221. **Table S2.** Overview of pathways identified via IPA and PANTHER. **Table S3.** Prediction of significantly correlated target genes of miR-24, miR-31, miR-106a, and miR-221. **Table S4.** Intra-individual mRNA and miRNA correlations. (XLSX 101 kb)
Additional file 2: Figures S1-S2.
**Figure S1**. Single tissue analysis of miRNA Expression in the human hair follicle. **Figure S2**. STRING query. (PDF 258 kb)

## Abbreviations

DPC: Dermal papilla cell
HF: Hair follicle
IPA: Ingenuity pathway analysis
miRNA/miR: MicroRNA
MPB: Male pattern baldness
PANTHER: Protein analysis through evolutionary relationship
PPI: Protein-protein interaction
STRING: Search tool for the retrieval of interacting genes/proteins
