# Supplementary material for: Expression profiling and bioinformatic analyses suggest new target genes and pathways for human hair follicle related microRNAs
Source: BMC Dermatol. 2017 Feb 22;17:3. doi: 10.1186/s12895-017-0054-9 (PMC5322611; doi:10.1186/s12895-017-0054-9)
Supplement: Additional file 2: Figures S1-S2. — Figure S1. Single tissue analysis of miRNA Expression in the human hair follicle. Figure S2. STRING query. (PDF 258 kb) [file 12895_2017_54_MOESM2_ESM.pdf]

Supplementary Figures:

Expression profiling and bioinformatic analyses suggests new target genes and pathways for human hair follicle related microRNAs

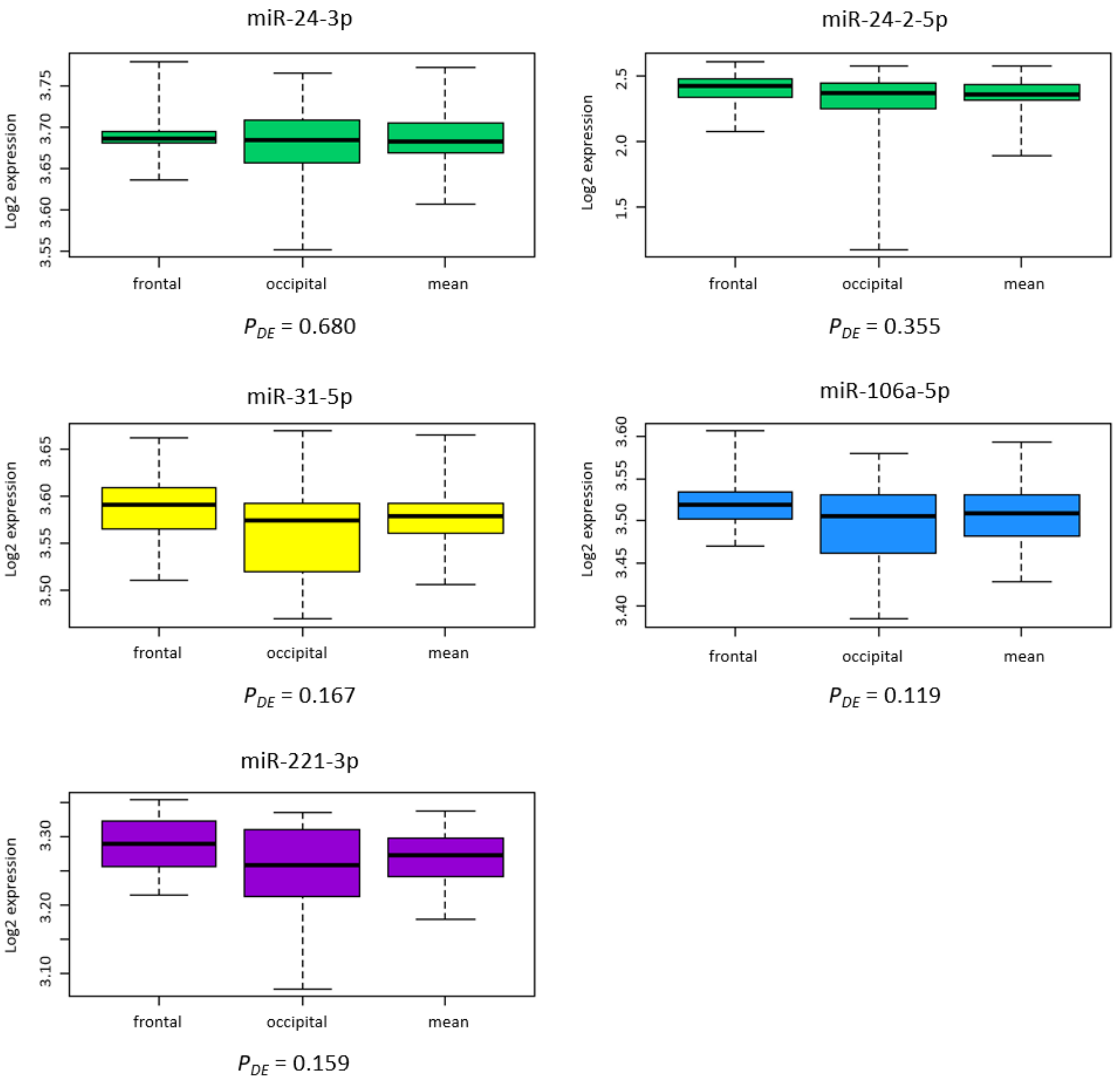

Supplementary Figure 1. **Single tissue analysis of miRNA Expression in the human hair follicle.** Boxplots representing frontal-, occipital- and the mean of frontal and occipital miRNA $\log_2$ expression in the human hair follicle. For those candidate miRNAs that show a significant mRNA correlation (miR-24, miR-31, miR106a, miR-221) no differential expression between hair follicles from the frontal and the occipital scalp areas is observed (Wilcoxon Rank-Sum Test, $P_{DE} < 0.001$ (Benjamini-Hochberg corrected)).

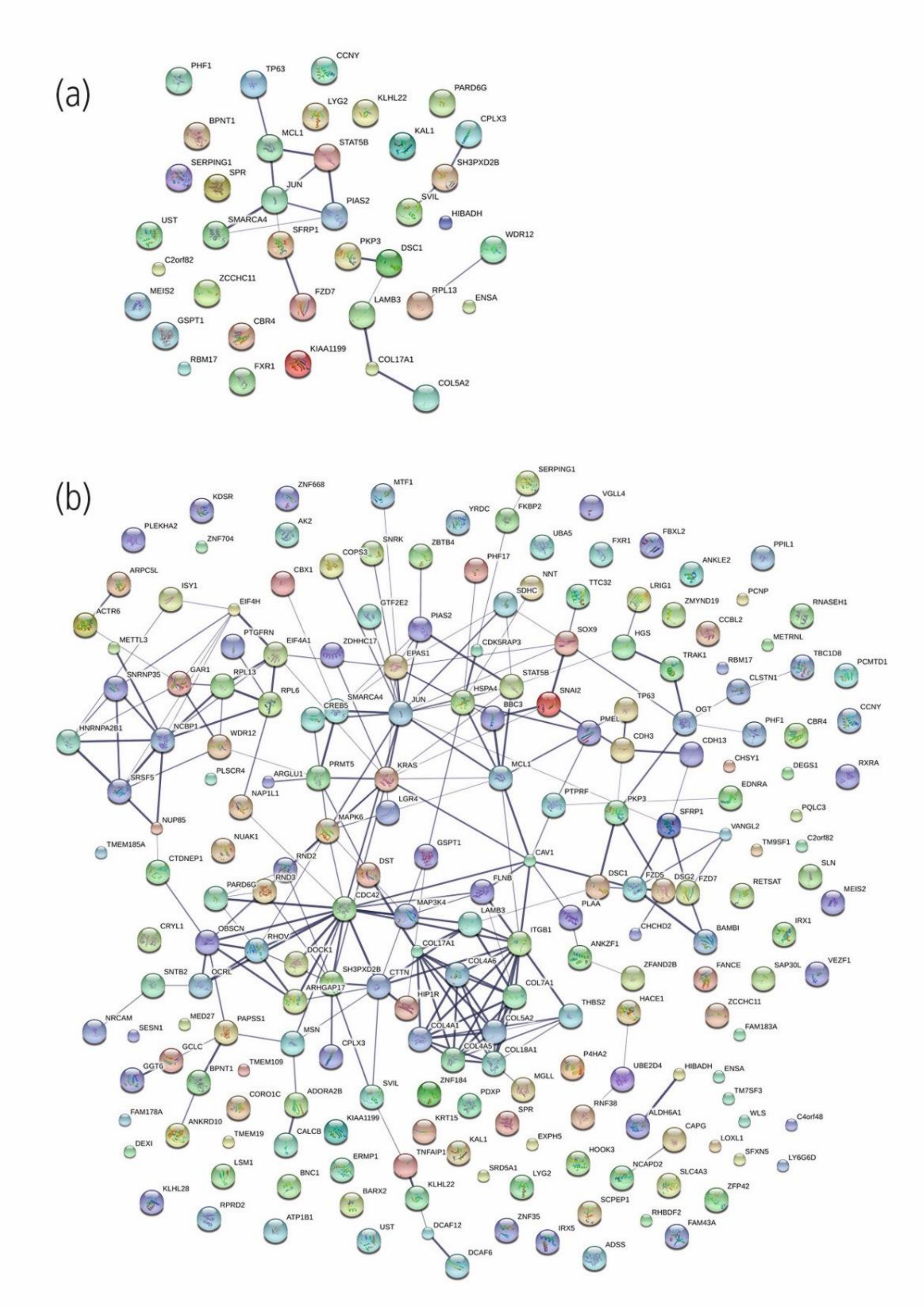

Supplementary Figure 2. **STRING query.** Protein-protein interaction network of: **(a)** the 40 shared target genes; and **(b)** all target genes of miR-24, miR-31, miR-106a and miR-221.
